# A chromosome-level genome assembly of the endangered rose *Rosa anemoniflora*

**DOI:** 10.1038/s41597-026-07516-5

**Published:** 2026-06-01

**Authors:** Xianzhen Zhou, Qun Yu, Chengjie Fu, Guojiang Lin, Xiaochun Huang, Tianqi Kang, Wei Gao

**Affiliations:** 1 Institute of Forest Ecology and Carbon Sink Measurement, Department of Forestry, Fujian Forestry Vocational and Technical College, Nanping, 353000 Fujian China; 2 Fujian Forestry Prospect and Design Institute, Fuzhou, 353001 Fujian China; 3 National Nature Reserve of Mangdang Mountain of Fujian Province, Nanping, 353000 Fujian China

## Abstract

*Rosa anemoniflora*, a critically endangered climbing rose endemic to China, holds significant ornamental and conservation value but lacks genomic resources. Here, we present a chromosome-level genome assembly generated using PacBio HiFi, Illumina, Iso-Seq, and Hi-C sequencing. The final assembly spans 547.52 Mb, with 95.72% of sequences anchored to seven pseudochromosomes and a contig N50 of 73.49 Mb. BUSCO assessment reveals 97.36% completeness (eudicotyledons_odb12). Repetitive elements constitute 55.31% of the genome, and 33,623 protein-coding genes were annotated, 97.68% of which are supported by BUSCO. We also identified 1,333 rRNA, 838 tRNA, and 1,043 other non-coding RNA genes. All data are publicly available at National Genomics Data Center (NGDC) (PRJCA052329). This high-quality genome provides a foundational resource for conservation genomics, evolutionary studies, and the genetic dissection of adaptive and reproductive traits in this threatened species.

## Background & Summary

The genus *Rosa* comprises approximately 200 species distributed widely across temperate to subtropical regions of Asia, Europe, and North America. Rosa species are characterized by complex ploidy levels ranging from diploid to decaploid and high heterozygosity, which together have fostered remarkable morphological and ecological diversity^1–3^. Cultivated roses have been extensively studied and utilized due to their significant economic value in the cut-flower, horticultural, and essential oil industries^4^. Meanwhile, wild rose resources—such as *R. roxburghii* and *R. davurica*—show great potential in functional foods and pharmaceuticals owing to their rich content of antioxidants and vitamin C^5–8^. In recent years, advances in high-throughput sequencing have led to chromosome-level genome assemblies for several cultivated and wild roses, such as *R. chinensis*^9^ and *R. gigantea*^10^, laying a foundation for evolutionary and functional genomics studies in the genus.

However, genomic information remains nearly absent for many wild rose species of unique biological value that are currently threatened or endangered. One such example is *R. anemoniflora* (Section *Synstylae*), a climbing shrub endemic to Nanping City, Fujian Province, China, growing at elevations of 400–1,000 m^11^. This species holds exceptional ornamental and germplasm value, with its vibrant flower color, elegant floral morphology, concentrated blooming period, and climbing habit making it highly promising for garden landscaping and urban greening. Naturally occurring on hillsides, wastelands, roadsides, and riverbanks within its elevation range, *R. anemoniflora* may possess genetic adaptations to environmental stresses such as drought and salinity-traits of potential relevance for breeding programs. Unfortunately, field surveys indicate extremely low population sizes, far below the minimum viable threshold for long-term persistence. In 2021, the species was listed as Near Threatened (NT) by the International Union for Conservation of Nature (IUCN) and included as a Class II protected species in China’s National Key Protected Wild Plants List (https://www.mee.gov.cn/xxgk2018/xxgk/xxgk01/202305/W020230522536560832337.pdf). Its critically low reproductive success (fruit set <15%, seed germination <20%) and high sensitivity to habitat fragmentation have exacerbated its conservation status^12,13^, creating an urgent need for genomic resources to inform evidence-based protection strategies.

To address this gap, we present the first chromosome-level, high-quality genome assembly of *R. anemoniflora*, generated by integrating PacBio HiFi^14^ long-read sequencing (~150 × coverage, based on the estimated genome size of ~547 Mb) and Hi-C^15^ chromatin conformation capture (~105 × coverage). The final assembly spans approximately 547.52 Mb, with a contig N50 of 73.49 Mb and a BUSCO completeness score of 97.29%. Hi-C scaffolding successfully anchored 95.72% of the assembly onto seven pseudochromosomes. All raw sequencing data, the assembled genome, and structural and functional annotation files have been deposited in public repositories at National Genomics Data Center (NGDC)^16^ with the accession number of PRJCA052329 in accordance with FAIR principles. This genomic resource fills a critical void in the genomics of endangered *Rosa* species. It provides a foundational platform for future research into the genetic mechanisms underlying *R. anemoniflora*’s endangered status, as well as for comparative genomics and conservation genetics studies in closely related taxa.

## Methods

### Sample collection and sequencing

The *R. anemoniflora* research material was collected from the Mangdangshan National Nature Reserve in Fujian (26°36'12” N, 118°02'30” E).

Genomic DNA was extracted from fresh leaves using the CTAB method. DNA integrity was assessed by electrophoresis on a 1% agarose gel stained with ethidium bromide. As shown in Supplementary Fig. 1, a single high-molecular-weight band (>15 kb) without smearing or RNA contamination was observed, indicating that the extracted DNA was of high integrity and suitable for downstream library construction. The DNA was used to construct Illumina short-read, PacBio HiFi long-read, and Hi-C chromatin conformation capture libraries.

Total RNA was extracted from the same leaf tissue using the RNeasy Plant Mini Kit (Qiagen) according to the manufacturer’s instructions. RNA integrity was assessed by electrophoresis on a 1% denaturing agarose gel stained with ethidium bromide. As shown in Supplementary Fig. 2, two clear bands corresponding to 28S rRNA and 18S rRNA were observed, with a 28S:18S intensity ratio of approximately 2:1 and no visible smearing, confirming high RNA integrity. cDNA was synthesized using the SMARTer PCR cDNA Synthesis Kit, size-selected (>1 kb) with BluePippin, and sequenced on a PacBio Sequel II platform, producing 4,782,271 CCS reads (N50 = 1,426 bp; GC content = 43.59%).

The Illumina library was sequenced on a NovaSeq. 6000 platform using paired-end 150 bp reads, yielding 72.89 Gb of raw data (~138 × genome coverage) for genome characterization. PacBio HiFi sequencing was performed on a Revio system, generating 28.72 Gb of high-fidelity long reads with an N50 read length of 20,455 bp. For the full-length transcriptome, cDNA was synthesized using the SMARTer PCR cDNA Synthesis Kit, size-selected (>1 kb) with BluePippin, and sequenced on a PacBio Sequel II platform, producing 4,782,271 CCS reads (N50 = 1,426 bp; GC content = 43.59%). The Hi-C library was sequenced on a DNBSEQ-T7 platform to support chromosome-scale scaffolding.

### Genome assembly

**Fig. 1:**
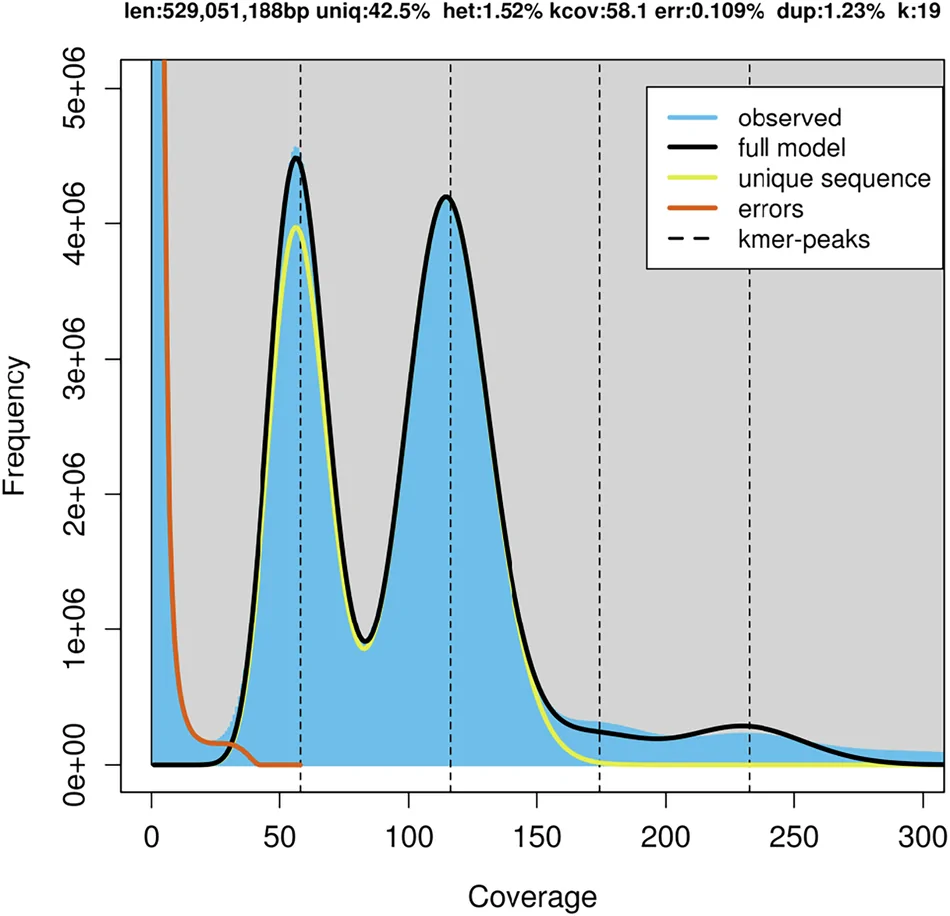
Distribution of k-mer frequencies employed to estimate the genome size.

**Fig. 2:**
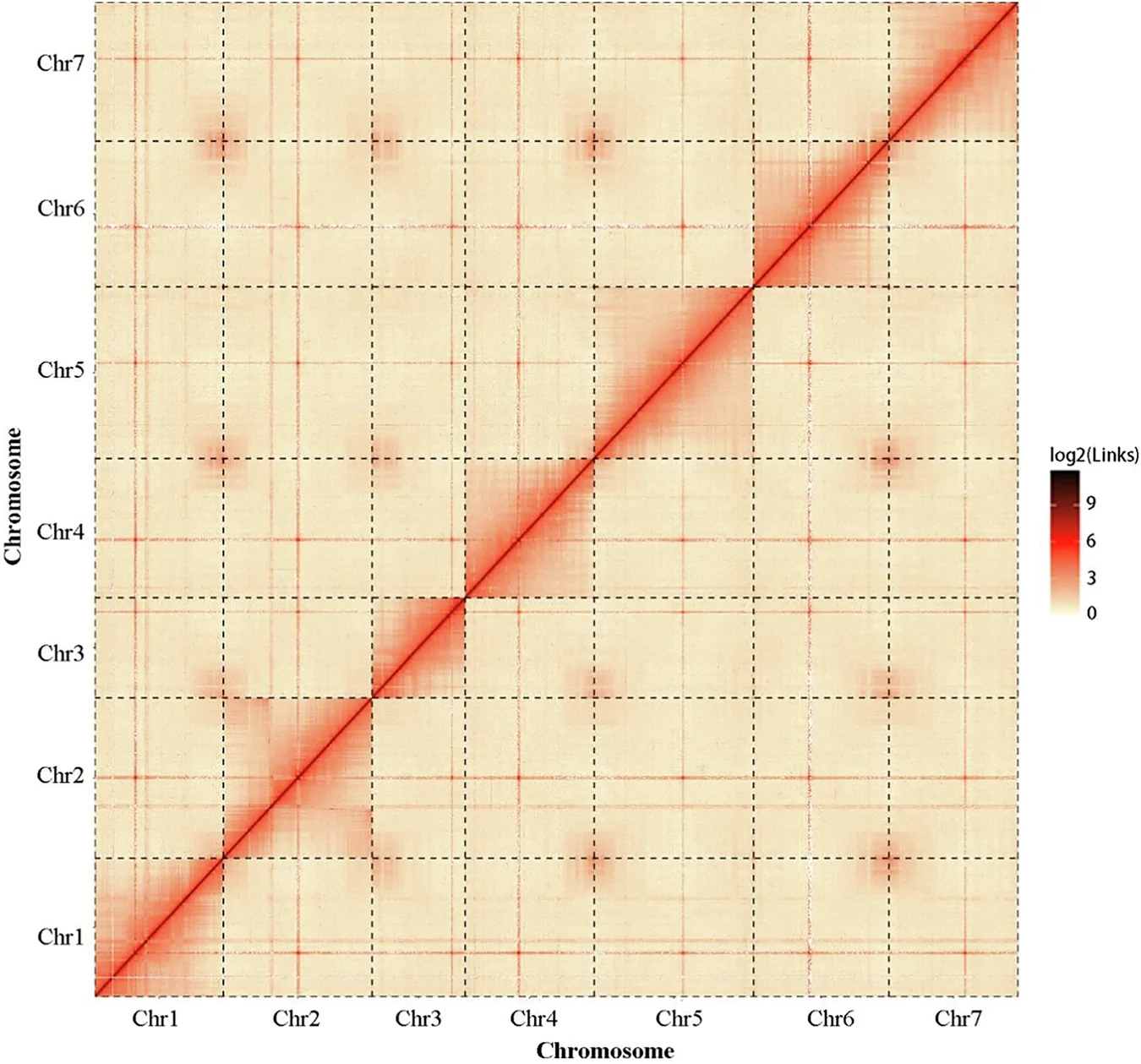
Hi-C intra-chromosomal contact map of the *R. anemoniflora* genome assembly.

Genome assembly followed a multi-step strategy. First, PacBio HiFi reads were assembled de novo using hifiasm^17^ v0.19.8 (default parameters, 32 threads) to generate an initial contig set. Redundant haplotigs were purged using purge_dups^18^ v1.2.5 based on k-mer depth distribution (main peak at 58.1 × ) and read coverage depth (Fig. 1). The cut-off value for haplotig identification was set to 70 (as estimated by the calcuts function), and sequences with coverage depth below this threshold were removed as haplotypic duplications, resulting in a haploid-resolved assembly (purged.fa) of 638.52 Mb across 128 scaffolds. After removing lowSubsequently, gene models containing frameshifts or coding sequence (CDS) lengths not divisible by three were filtered out to ensure the integrity of protein-coding sequence-quality and short sequences, a haploid-resolved assembly (draft.fa) of 542.26 Mb across 109 contigs was retained for subsequent Hi-C scaffolding. Hi-C data were then integrated using 3D-DNA^19^ v201008 with default parameters with 2 rounds of misassembly correction, -r 2) to anchor contigs into chromosome-scale pseudomolecules, followed by manual correction with Juicebox Assembly Tools. The final assembly spans 547.52 Mb with a contig N50 of 73.49 Mb, and 534.43 Mb (95.72%) was anchored to seven pseudochromosomes (Table 1 and Fig. 2), corresponding to the haploid chromosome number of this diploid species (2n = 2x = 14). Assembly completeness was assessed with BUSCO^20^ v6.0.0 using the eudicotyledons_odb12 (2,805 genes): 97.36% of BUSCOs were complete (Table 1), 0.82% were fragmented, and 1.82% were missing. Additionally, Illumina short reads from the genome survey were remapped to the final assembly using BWA-MEM^21^ v0.7.17 (default settings), achieving a mapping rate of 98.10% and a coverage breadth of 99.27%.

**Table 1:** Basic statistics of the *Rosa anemoniflora* genome assembly and annotation.

Item	Value
Total assembly size (bp)	547,519,537
Number of chromosomes anchored	7
Proportion of sequence anchored to chromosomes	0.9572
Contig N50 (bp)	73,487,902
GC content (%)	38.75
BUSCO completeness (%)	97.36
Total number of genes	33,623
Total length of genic regions (bp)	117,246,647
Proportion of genic regions in the genome (%)	21.41
Average gene length (bp)	3,487
Average number of exons per gene	4.8
Average exon length (bp)	238.6
Average intron length (bp)	603.5

### Genome annotation

Repeat elements were annotated using a combined approach: a species-specific repeat library was constructed with RepeatModeler^22^ v2.0.6 (https://github.com/Dfam-consortium/TETools), supplemented with RepBase^23^, and applied via RepeatMasker^24^ v4.1.2. LTR retrotransposons were further refined using EDTA^25^ v2.2.2 for structural prediction and classification. Repeats constitute 55.31% of the genome, dominated by LTR retrotransposons (31.48%), followed by unclassified repeats (13.92%), DNA transposons (7.10%), LINEs (2.81%), and SINEs (0.00%) (Table 2).

**Table 2:** Repetitive element composition in the *Rosa anemoniflora* genome.

Repeat Class	Repeat Size (bp)	Percentage of Genome (%)
LTR retrotransposons	172,344,069	31.48
DNA transposons	38,855,010	7.1
LINEs	15,411,239	2.81
SINEs	2,692	0
Penelope	41,541	0.01
Retroviral	233,424	0.04
Unclassified	76,235,085	13.92
Satellites	781,359	0.14
Simple repeats	134,576	0.02
Total interspersed repeats	302,848,095	55.31

**Fig. 3:**
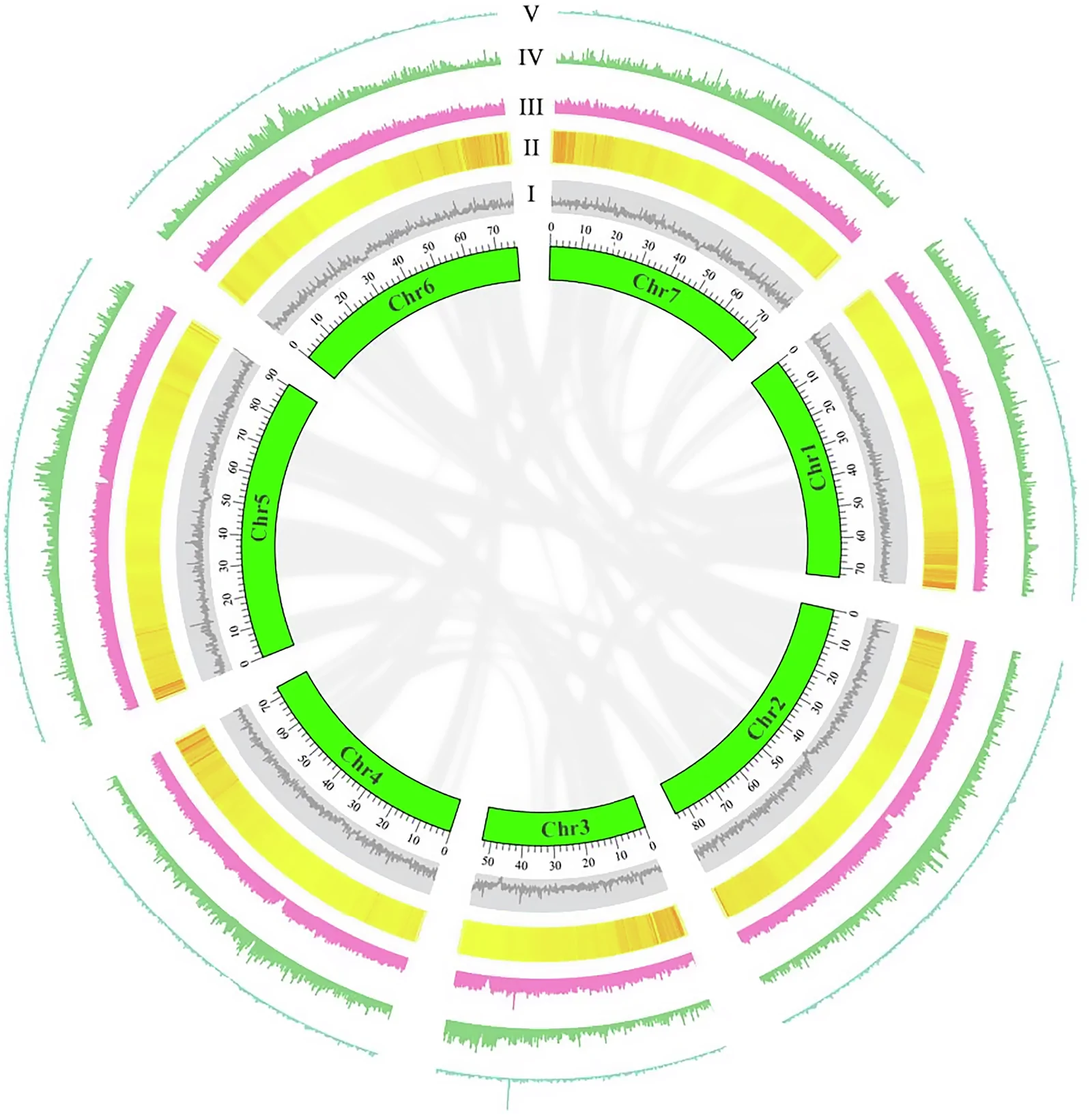
Genome landscape of *Rosa anemoniflora*. Tracks from outer to inner circles: (I) GC content (%) in 100-kb windows; (II) gene density (number of genes per 100-kb window); (III) repeat density (number of repetitive sequences per 100-kb window); (IV) density of LTR/Gypsy elements; (V) density of LTR/Copia elements. Central lines indicate intra-genomic collinear blocks identified by MCScanX.

Protein-coding genes were predicted by integrating *ab initio*, homology-based, and transcriptome evidence. *Ab initio* predictions were generated with Augustus^26^ v3.3.2 and GeneMark-ES^27^ v4.71. Homology-based annotation leveraged protein sequences from *Arabidopsis thaliana*, *Fragaria vesca*, and *Rosa chinensis* using GeMoMa^28^ v1.9 and exonerate^29^ v2.2.0. Transcriptomic evidence came from PacBio Iso-Seq CCS reads processed with PASA^30^ v2.5.2. These three lines of evidence were weighted (homology: 10, transcriptome: 12, ab initio: 4) and merged using EVidenceModeler^31^ v1.1.1 to produce a high-confidence consensus gene set. Subsequently, gene models containing frameshifts or coding sequence (CDS) lengths not divisible by three were filtered out to ensure the integrity of protein-coding sequences. A total of 33,623 protein-coding genes were annotated, with an average of 4.8 exons per gene and a mean gene length of 3,487 bp (Table 1). BUSCO analysis of the gene set recovered 97.68% of conserved eudicot genes as complete. Functional annotation was performed by aligning proteins to NR and TrEMBL^32^ databases using DIAMOND^33^ v2.0.14 (e-value ≤ 1e−5) and identifying domains with InterProScan^34^ v5.50–84.0, enabling mapping to KEGG^35^ and Gene Ontology^36^ (GO) categories. Genomic features were visualized using Circos^37^ (Fig. 3).

Non-coding RNAs were identified as follows: tRNAs were predicted with tRNAscan-SE^38^ v1.3.1; rRNA genes (5S, 18S, 28S) were annotated using RNAmmer^39^ v1.2; other ncRNAs were detected by homology search against Rfam^40^ v1.0. Annotation statistics are summarized in Table 3.

**Table 3:** Statistics of non-coding RNA prediction results.

ncRNA Type	Copy	Avg. length (bp)	Total length (bp)	% of genome
5s_rRNA	563	115.23	64,872	0.0118
18s_rRNA	383	1792.3	686,449	0.1254
28s_rRNA	387	3,864.67	1,495,629	0.2732
tRNA	838	74.29	62,254	0.0114
other ncRNA	1,043	132.49	138,190	0.0252

## Data Records

The raw sequencing data has been deposited in the Genome Sequence Archive (GSA) at the China National Center for Bioinformation (CNCB; Project Accession: PRJCA052329). The Hi-C reads are available under GSA accession CRA034365 (Run ID: CRR2373096)^41^. The final genome assembly (in FASTA format) has been deposited in NCBI GenBank under accession number GCA_054131425.1^42^ Gene structural annotations (in GFF3 format) and functional annotation files are publicly available on Figshare^43^.

## Technical Validation

The quality of the *R. anemoniflora* genome assembly and annotation was evaluated using multiple complementary approaches.

Assembly contiguity and completeness. The final assembly achieved high contiguity, with a contig N50 of 73.49 Mb, an N90 of 52.83 Mb, and the longest sequence reaching 90.62 Mb. A total of 95.72% of the assembly (534.43 Mb) was successfully anchored to seven pseudochromosomes, corresponding to the haploid chromosome number of this diploid species (2n = 2x = 14). Benchmarking Universal Single-Copy Orthologs (BUSCO) analysis against the eudicotyledons_odb12 (2,805 genes) recovered 97.36% complete genes (93.90% single-copy, 3.46% duplicated), with only 0.82% fragmented and 1.82% missing, indicating near-complete representation of the expected gene space. As an additional validation, Compleasm^44^ analysis using the more recent eudicotyledons_odb12 lineage (2,805 genes) yielded 99.08% complete BUSCOs (95.19% single-copy, 3.89% duplicated), further confirming the high completeness of the assembly.

Annotation validation. The gene annotation pipeline integrated *ab initio*, homology-based, and transcriptome evidence, yielding 33,623 protein-coding genes. BUSCO assessment of the gene set recovered 97.68% complete BUSCOs (93.58% single-copy, 4.10% duplicated), with 1.00% fragmented genes and only 1.32% missing, demonstrating high annotation completeness. Functional annotation assigned putative functions to 95.90% of the predicted genes across major public databases (NR, Swiss-Prot, Pfam, GO, KEGG), providing a valuable resource for downstream functional studies.

Together, these multi-faceted validation results confirm that the *R. anemoniflora* genome assembly and annotation are of high quality and suitable for conservation genomics, evolutionary analyses, and comparative genomic studies within the genus *Rosa*.

## Supplementary information


Supplementary Information


## Data Availability

This dataset has not been previously published or presented, and no conflicts of interest are declared. The raw sequencing reads and chromosome-scale genome assembly of *R. anemoniflora* have been deposited in the National Genomics Data Center (NGDC) as described in Data Records. Additionally, annotation data have been deposited in the figshare database (https://doi.org/10.6084/m9.figshare.30742499.v1).

## Code availability

All analyses were performed strictly in accordance with the manuals and protocols of the publicly available tools, and no custom code was utilized in this study.
