# Supplementary material for: A chromosome-level genome assembly of the endangered rose Rosa anemoniflora
Source: Sci Data. 2026 Jun 1;13:1238. doi: 10.1038/s41597-026-07516-5 (PMC13521891; doi:10.1038/s41597-026-07516-5)
Supplement: Supplementary file 1 [file 41597_2026_7516_MOESM1_ESM.pdf]

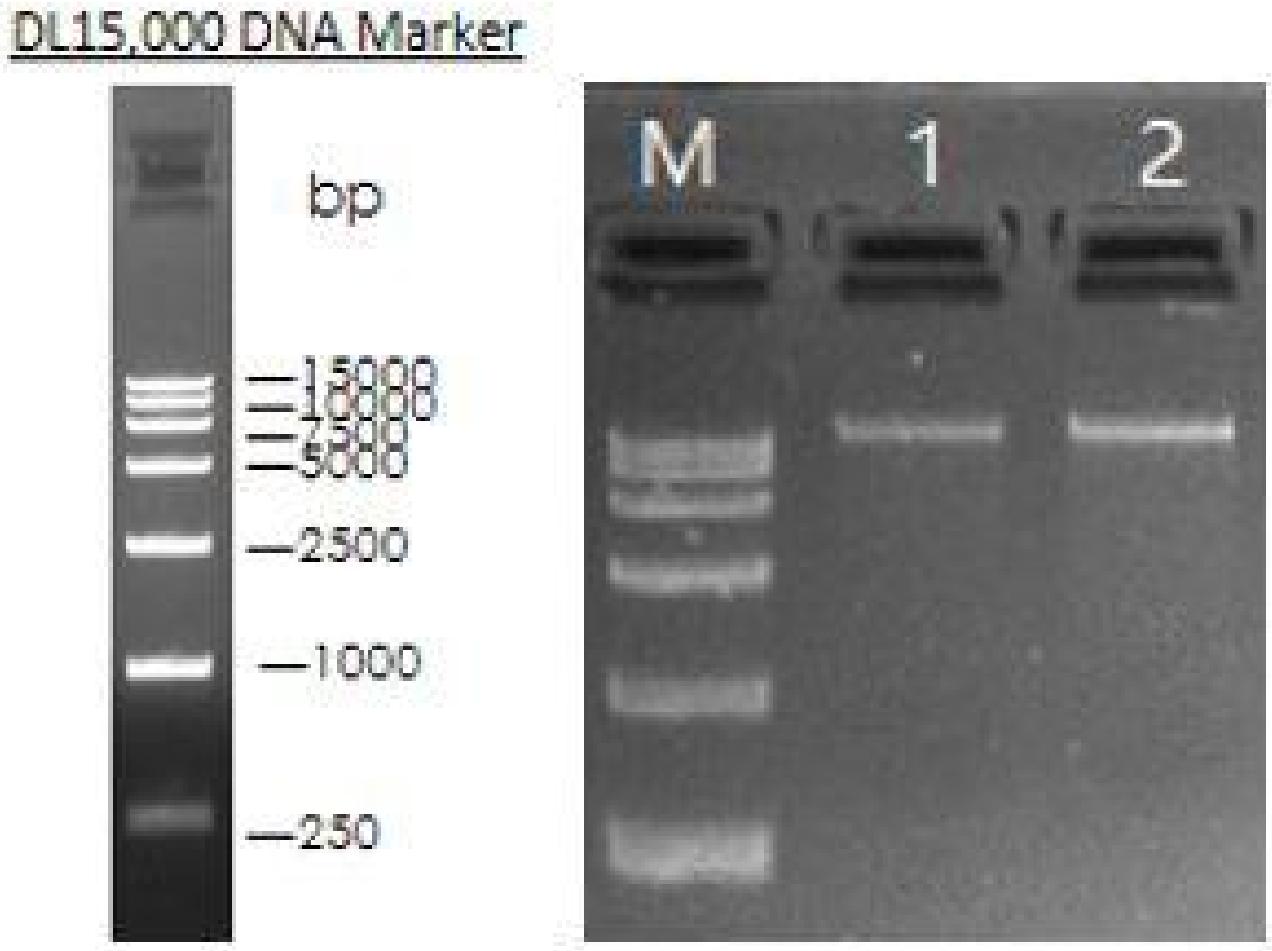

Figure S1 Agarose gel electrophoresis of genomic DNA extracted from *R. anemoniflora*. Lane M: DL15,000 DNA marker (band sizes: 15,000, 8,000, 5,000, 2,500, 1,000, 250 bp). Lanes 1–2: Genomic DNA samples. A single high-molecular-weight band (>15 kb) without smearing indicates high DNA integrity and absence of significant degradation or RNA contamination.

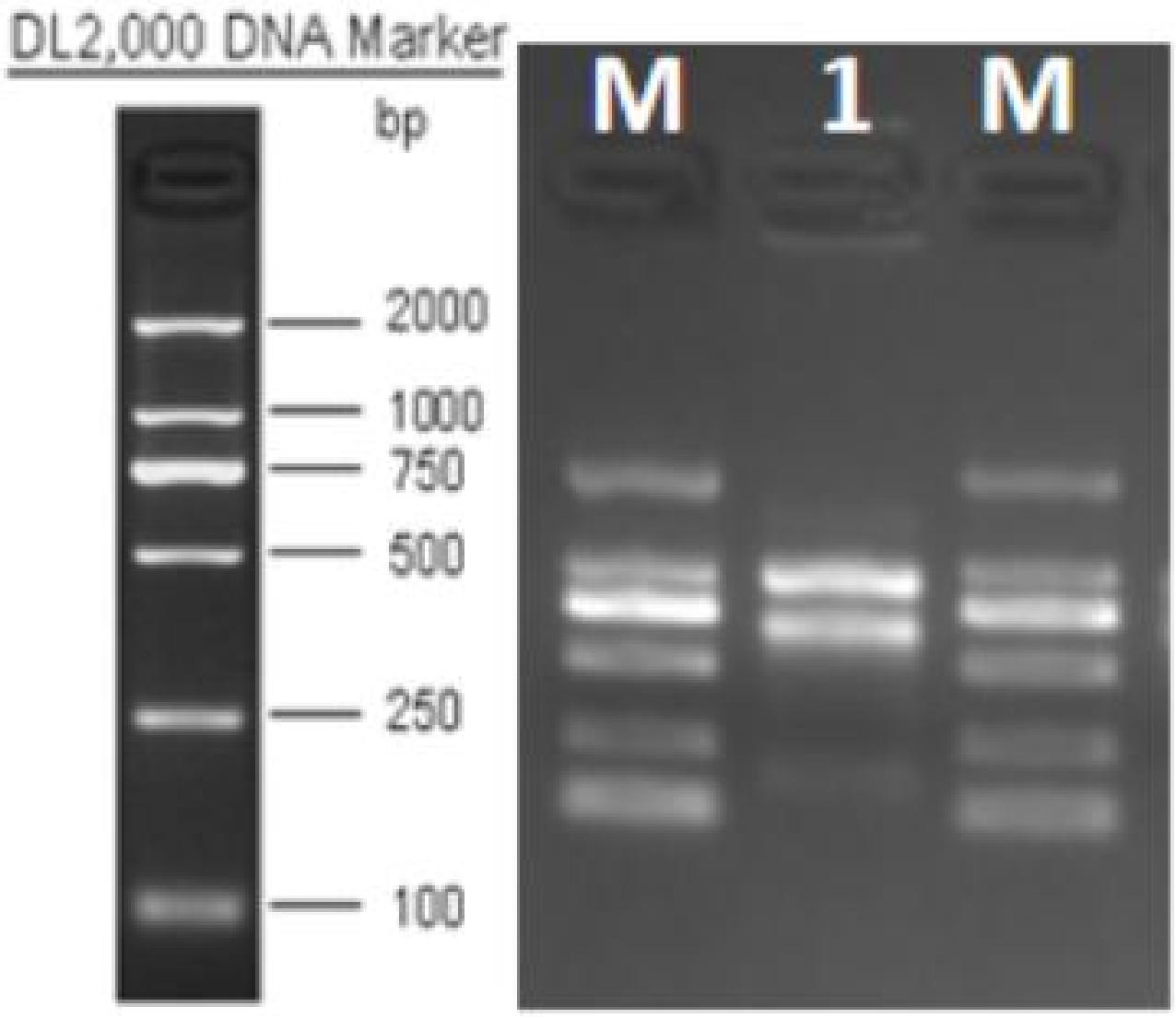

Figure S2 Agarose gel electrophoresis of total RNA extracted from *R. anemoniflora* leaves. Lane M: DL2,000 DNA marker (band sizes: 2000, 1000, 750, 500, 250, 100 bp). Lanes 1 – 2: Two independent RNA samples. Both samples show two distinct bands corresponding to 28S rRNA (~5 kb) and 18S rRNA (~2 kb), with a 28S:18S ratio of approximately 2:1, indicating high RNA integrity and absence of degradation or DNA contamination.
